# Predictive factors of alcohol and tobacco use in adolescents

**DOI:** 10.1590/0104-1169.3570.2516

**Published:** 2014

**Authors:** Alicia Alvarez-Aguirre, María Magdalena Alonso-Castillo, Ana Carolina Guidorizzi Zanetti

**Affiliations:** 1 PhD, Professor, Facultad de Enfermería, Universidad Autónoma de Querétaro, Santiago de Querétaro, Mexico; 2 PhD, Professor, Facultad de Enfermería, Universidad Autónoma de Nuevo León, Nuevo León, Mexico; 3 PhD, Professor, Escola de Enfermagem de Ribeirão Preto, Universidade de São Paulo, WHO Collaborating Centre for Nursing Research Development, Ribeirão Preto, SP, Brazil

**Keywords:** Self Concept, Assertiveness, Self Efficacy, Resilience, Psychological

## Abstract

**OBJECTIVES:**

to analyze the effect of self-esteem, assertiveness, self-efficacy and resiliency on alcohol and tobacco consumption in adolescents.

**METHOD:**

a descriptive and correlational study was undertaken with 575 adolescents in 2010. The Self-Esteem Scale, the Situational Confidence Scale, the Assertiveness Questionnaire and the Resiliency Scale were used.

**RESULTS:**

the adjustment of the logistic regression model, considering age, sex, self-esteem, assertiveness, self-efficacy and resiliency, demonstrates significance in the consumption of alcohol and tobacco. Age, resiliency and assertiveness predict alcohol consumption in the lifetime and assertiveness predicts alcohol consumption in the last year. Similarly, age and sex predict tobacco consumption in the lifetime and age in the last year.

**CONCLUSION:**

this study can offer important information to plan nursing interventions involving adolescent alcohol and tobacco users.

## Introduction

Alcohol and tobacco consumption is a concern of health systems. These psychoactive substances reveal the highest consumption rates in the Mexican population. They are considered initial drugs and one of the their negative effects is the enhanced risk of illegal drug use. In addition, in different Brazilian and international addiction studies and surveys, it is registered that the proportion of adolescents who consume drugs, mainly including alcohol and tobacco, increases progressively and that consumption starts before the age of 18 years^(^ 1 ^-^ 2 ^)^.

Furthermore, adolescent problems like violence, accidents, interpersonal difficulties, low competence and school drop-out are related to the early onset of alcohol and tobacco consumption^(^ 3 ^)^. On the other hand, the authors report that the usage pattern of these drugs is heterogeneous and ranges from experimenting to dependence^(^ 4 ^)^.

In that sense, the adolescents with greater probability of using drugs like alcohol and tobacco are exposed to different risk factors, related to personal factors, the adolescents' development context and their cognition related to health promotion^(^ 4 ^)^.

As documented in the literature, the adolescents are particularly vulnerable to damage in their development and physical integrity when they consume drugs like alcohol and tobacco. Therefore, in this group, the personal factors and thoughts on health promotion and prevention of alcohol and tobacco consumption need to be identified, factors that can protect the adolescents from the start of drug consumption, use and abuse^(^ 5 ^-^ 6 ^)^.

For this study, the personal factors include the characteristics that make people unique or distinguish them. These factors are classified as biological, sociocultural and psychological. Some of the personal factors cannot be modified. In this respect, self-esteem was considered as a psychological personal factor and age and gender as biological factors. On the other hand, the thoughts related to the conduct of not consuming alcohol and tobacco are assertiveness, self-efficacy and resiliency. These variables are modifiable objectives, due to their strong motivational influence on health conducts and interventions^(^ 7 ^)^.

This study intends to contribute to the knowledge on this phenomenon based on the analysis of the effect of personal factors and cognitions on the conduct of non-consumption of alcohol and tobacco in adolescents living in the rural area who study at secondary schools in a city in the state of Guanajuato, Mexico. Therefore, the study objectives were to: 1) Describe the prevalence of alcohol and tobacco consumption in the lifetime and in the last year in secondary students in the rural area. 2) Describe the self-esteem, assertiveness, self-efficacy and resiliency in secondary students per gender, school year and occupation. 3) Report on the effect of self-esteem, assertiveness, self-efficacy and resiliency on alcohol and tobacco consumption in the lifetime and in the last year.

## Method

Quantitative and descriptive correlation study^(^ 8 ^)^. The participants were assessed on a single occasion. The data were collected at 14 public secondary schools in a city in the state of Guanajuato, Mexico, during two weeks in November 2010. The study population consisted of adolescents between 12 and 18 years of age, enrolled in the school year 2010-2011. Temporal sampling was used and the sample consisted of all adolescents (census) who accepted to participated, whose parents or responsible caregivers signed their consent to participate in the study and who, in addition, were present at the time of the data collection (*n*=575). Before collecting the data, the study objectives were explained, emphasizing the participants' anonymity and the confidentiality of the information. Next, the participants completed a form with personal data (age, sex, school year and occupation) and the prevalence history of drug consumption (consumption in the lifetime and in the last year). In the data collection, four measuring instruments were used: Self-Esteem Scale^(^ 7 ^)^, the Situational Confidence Scale^(^ 8 ^)^, the Assertiveness Questionnaire^(^ 9 ^)^ and the Resiliency Scale^(^ 10 ^)^, instruments that have been applied in the Mexican population with acceptable Cronbach's alpha coefficients. In this case, the internal consistency of the instruments was as follows: Self-Esteem Scale (α=.66), Situational Confidence Scale (α=.99), Assertiveness Questionnaire (α=.66) and Resiliency Scale (α=.98).

In the data collection, besides the primary author, seven research assistants participated who had been previously trained for this purpose. Using posters and personal invitations, classroom per classroom, all adolescents were summoned to participate in the study. Interested students remained in the classroom for 60 minutes to sign their consent and complete the instruments. Each participant received a yellow envelope with the documents. When they returned the instruments, they were asked whether they had completed all questionnaires and questions. At the end of the data collection, the participant placed the envelope in a box located in the same classroom.

To analyze the information, a database was produced in the statistical software for the social sciences - SPSS, version 17. The internal consistency of each of the instruments was assessed with the help of Cronbach's alpha coefficient. Frequencies, proportions and percentages were obtained for the categorical variables. For the numerical variables, distribution, central trend and variation measures were calculated. Kolmogorov-Smirnov's Goodness-of-Fit test was applied with Lilliefors' correction to contrast the normality hypothesis in the distribution of the continuous variables, showing significance in the response variables (p<.01). Therefore, the use of non-parametric or free distribution tests was chosen. With regard to objective one, contingency tables were used through frequencies and proportions and point estimates were calculated with a 95% confidence interval. For objective two, indices were elaborated and hypotheses were contrasted using Mann-Whitney's U-test and Kruskal-Wallis' H-test. For objective three, the Logistic Regression Model was used.

Approval for this study was obtained from the ethics committee at the School of Nursing of Universidad Autónoma de Nuevo León on June 30^th^ 2010, registration number FAEN-D-753.

## Results

With regard to the participants' sociodemographic and occupational characteristics, it was observed that 56.9 % was between 12 and 13 years of age. Concerning the sex, girls were predominant (51.5%). Regarding the school year, 44.2 % were first-year students and 22.3 % of the students worked.

The students' mean age was 13.3 years (*SD*=1.08). For alcohol consumption, the mean age when alcohol consumption started was 11.6 years (*SD*=1.49), with a mean consumption of 1.7 alcoholic beverages on a typical day (*SD*=1.0). Concerning tobacco consumption, the mean age when this consumption started was 11.9 years (*SD*=1.43) and, on average, they consumed one cigarette (Mean=1.60, *SD*=1.61) on a typical day.

In response to the first objective, which is to describe the prevalence of alcohol and tobacco consumption in the lifetime and in the last year in rural secondary students, contingency tables were used through frequencies and proportions. In addition, point estimates were calculated with a 95% confidence interval. In this respect, the results showed that 66.1% (95% CI [62%-70%]) of the rural secondary students had consumed alcohol in the lifetime and that 32.2% (95% CI [28%-36%]) did so in the last year. On the other hand, 30.3% (95% CI [26%-34%]) of the participants reported tobacco consumption in the lifetime and 13.6% (95% CI [11%-16%]) in the last year.

In response to the second objective, which is to describe the self-esteem, assertiveness, self-efficacy and resiliency in secondary students per gender, school year and occupation, indices were elaborated and hypotheses were contrasted using Mann-Whitney's U-test and Kruskal-Wallis' H-test.

The results showed that there is a significant difference between self-efficacy (*U*=35061.00, *p=*.002), assertiveness (*U*=32690.00, *p<*.001) and resiliency (*U*=35559.50, *p=*.004) per gender (Table 1). Nevertheless, self-esteem showed no significant difference (*p*>.05). The women displayed higher medians for self-efficacy (*Mdn*=66.67), assertiveness (*Mdn*=42.24) and resiliency (*Mdn*=77.14) when compared to men (*Mdn*=45.73, *Mdn*=38.79 and *Mdn*=72.57 respectively). The median self-esteem was the same for men and women (*Mdn*=47.50).

**Table 1 -:** Mann-Whitney's U-test for Self-Esteem, Self- Efficacy, Assertiveness and Resiliency per Gender. Guanajuato, Mexico, 2014 (N=575)

Sex		Mean	Mdn	Mann-Whitney’s U-test statistics	p-value*
Self-esteem				39812.50	0.45
	Male	48.38	47.50		
	Female	49.17	47.50		
Self-efficacy				35061.00	0.002
	Male	46.91	45.73		
	Female	52.54	66.67		
Assertiveness				32690.00	0.001
	Male	39.85	38.79		
	Female	42.12	42.24		
Resiliency				35559.50	0.004
	Male	61.07	72.57		
	Female	68.94	77.14		

* p<0.01

As regards the school year, the results showed a significant difference in self-esteem (*H*=14.89, *p<*.001), assertiveness (*H*=7.99, *p<*.05) and resiliency (*H*=12.10, *p<*.05); no significant difference was found between self-efficacy and school year (*p*>.05). The third-year students showed higher medians for self-esteem, assertiveness, self-efficacy and resiliency: self-esteem (*Mdn*=50.0), self-efficacy (*Mdn*=66.67), assertiveness (*Mdn*=42.24) and resiliency (*Mdn*=79.14).

Similarly, the results showed a significant difference in assertiveness (*U*=22942.50, *p<*.001) according to the occupation level. The remaining thoughts related to the non-consumption of alcohol and tobacco showed no significant difference: self-esteem (*p*>.05), self-efficacy (*p*>.05) and resiliency (*p*>.05) according to different occupation levels. Students who do not work presented higher median assertiveness (*Mdn*=41.38).

In response to the third objective, which is to report on the effect of self-esteem, assertiveness, self-efficacy and resiliency on alcohol consumption in the lifetime and in the last year, the Logistic Regression Model was used. To discard the collinearity between the independent variables of the regression model, Pearson's correlation test was used for the continuing variables (age, self-esteem, assertiveness, resiliency and self-efficacy) and Cramer's V correlation when any of the variables was dichotomous (gender).

In the first case (Pearson's correlation), the identified coefficients showed a weak to moderate correlation, in which the highest coefficient (0.48) corresponds to the correlation between self-esteem and assertiveness (Table 2).

**Table 2 -:** Pearson's correlation. Guanajuato, Mexico, 2014

Variables		r*	P-value†
Age			
	Self-esteem	0,07	0,09
	Assertiveness	-0,01	0,78
	Resiliency	0,11	0,01
	Self-efficacy	0,06	0,14
Self-esteem			
	Assertiveness	0,48	0,00
	Resiliency	0,34	0,00
	Self-efficacy	0,23	0,00
Assertiveness			
	Resiliency	0,34	0,00
	Self-efficacy	0,16	0,00
Resiliency			
	Self-efficacy	0,33	0,00

* Pearson's Correlation

† p<0.01

The same scenario appears for the dichotomous variable gender, although Cramer's V correlation coefficients in this analysis correspond to 0.55 for gender and self-efficacy (Table 3).

**Table 3 -:** Cramer's V correlation, Mexico, 2014

Variables		r*
Sex		
	Age	0.14
	Self-esteem	0.23
	Assertiveness	0.35
	Resiliency	0.45
	Self-efficacy	0.55

* Pearson's Correlation

The co-linearity was assessed based on the statistical significance (inside the regression model) of the independent variables in each of the regression models. Four models were proposed that were significant in general but, when considering the particular level, the independent variables were not significant. In this case, in each of the models, the independent variables without statistical significance were gradually eliminated until finding the model with the best general fit, and which showed statistical significance for the independent variables at the particular level.

Table 4 presents the adjustment of two logistic regression models. In the first, the independent variables age, assertiveness and resiliency were considered, which shows a significant effect on alcohol consumption in the lifetime (*X* *2*=44.52, *gl*=3,* p<*.001), corresponding to 7.5% of explained variance. In the second model, the independent variables age and assertiveness were included, demonstrating a significant effect on alcohol consumption in the last year (*X* *2*=28.54, *gl*=2,* p<*.001) and corresponding to 4.8% of explained variance.

**Table 4 -:** Effect of independent variables on alcohol consumption in the lifetime and in the last year. Guanajuato, Mexico, 2014 (N=575)

Variables		Estimates of B coefficients	Standard deviation	Wald statistics	Degrees of freedom	P value *
Consumption in the lifetime						
	Age	0.474	0.093	26.296	1	0.001
	Assertiveness	0.028	0.014	4.104	1	0.043
	Resiliency	0.008	0.004	4.813	1	0.028
Consumption in the last year						
	Age	0.379	0.085	19.68	1	0.001
	Assertiveness	0.038	0.013	8.60	1	0.001

* p<0.01

As observed, the variables that are able to predict the probability of alcohol consumption in the lifetime were age, assertiveness and resiliency. Furthermore, the variables that can predict the probability of alcohol consumption in the last year are age and assertiveness.

In Table 5, the adjustment of two logistic regression models is presented. In the first, the independent variables age and gender were considered, who shows a significant effect on tobacco consumption in the lifetime (*X* *2*=60.23, *gl*=2,* p<*.001), corresponding to 9.9% of explained variance. The second considered the independent variable age, which shows a significant effect on tobacco consumption in the last year (*X* *2*=16.86, *gl*=1,* p<*.001), corresponding to 2.9% of explained variance.

**Table 5 -:** Effect of independent variables on tobacco consumption. Guanajuato, Mexico, 2014 (N=575)

Variables		Estimates of B coefficients	Standard deviation	Wald statistics	Degrees of freedom	P value*
Consumption in the lifetime						
	Sex	-0.709	0.19	12.84	1	0.001
	Age	0.582	0.09	39.07	1	0.001
Consumption in the last year						
	Age	0.452	0.111	16.61	1	0.001

* p<0.01

As observed, the variables that can predict the probability of tobacco consumption in the lifetime are age and gender. Similarly, the variable that can predict the probability of tobacco consumption in the last year is age.

## Discussion

The sample consisted of 575 adolescent students, with ages ranging between 12 and 18 years. In the sociodemographic profile for this study, a higher proportion of girls was found; a greater proportion of students were in the first year; a small proportion of the participants studies and works. These results are in line with the findings from institutions^(^ 11 ^)^, showing a larger proportion of women than men and a small proportion of working students.

The adolescents started their consumption of alcohol and tobacco at the age of 11 years, differently from the findings in Brazilian surveys^(^ 2 ^)^. Adolescence is a critical period, characterized by changes and adaptations, in which people try to achieve autonomy, acquire skills and experience new sensations^(^ 12 ^-^ 13 ^)^.

With regards to the students' thoughts on the non-consumption of alcohol and tobacco according to gender, school year and occupation, the results showed a significant difference in the variables self-efficacy, assertiveness and resiliency according to gender, with women showing higher medians for self-efficacy, assertiveness and resiliency when compared to men. Nevertheless, the median self-esteem was the same for men and women. The gender role probably exerts influence in the case of women, in that the sociocultural premises assign a protective role to women, which implies that women consider they have greater strength and resistance (self-efficacy and resiliency) to cope with the problems that emerge^(^ 14 ^-^ 15 ^)^.

What the school year is concerned, the results revealed a significant difference in self-esteem, assertiveness and resiliency. The third-year students higher median self-esteem, assertiveness, self-efficacy and resiliency, probably related to the result of their education, given that they are almost graduating from secondary school and that, for many, this is their final education opportunity. In many cases, this is the highest educational level achieved in their family, which makes them feel strong and resilient to cope with any problem in their experiences^(^ 16 ^)^.

Similarly, the results indicated a significant difference in assertiveness according to the occupation level. The students who worked showed higher median self-efficacy, which can be due to the certain degree of independence that results from gaining one's own revenues. On the other hand, the students who did not work showed higher median assertiveness.

Finally, it was shown that the variables age, resiliency and assertiveness predict alcohol consumption in the lifetime and that the variables age and assertiveness predict alcohol consumption in the last years. Similarly, age and gender predict tobacco consumption in the lifetime and age in the last year. These findings are in line with different authors^(^ 13 ^,^ 16 ^-^ 18 ^)^, who contribute to the concept of resiliency and assertiveness, in the sense that the ability to cope with different difficulties in their context, as well as the ability to say no to alcohol consumption, can serve as a protection factor. In addition, another research reported that alcohol consumption can vary in function of age and sex^(^ 18 ^)^, which is why further research is needed on the factors related to drug consumption from a nursing perspective, as the nurse plays a fundamental role to propose and implement educational interventions on this theme.

It should be highlighted that the effect of self-esteem and self-efficacy on the consumption of these substances in the lifetime was not observed, as opposed to the findings in different studies, probably because the sample was obtained in the rural context^(^ 17 ^-^ 18 ^)^. This fact is probably due to the low and homogeneous self-esteem and self-efficacy scores.

## Conclusion

This study describes the prevalence of alcohol and tobacco consumption in the lifetime and in the last year and the personal factors for the aspects evaluated in the 575 rural secondary students. In addition, the effect of these factors on consumption was presented.

The importance of further longitudinal and prospective research on self-esteem and thoughts related to the conduct of not consuming alcohol and tobacco should be highlighted. Furthermore, the scales applied in this study should be used further, given the acceptable internal consistency that was demonstrated.

This study can provide important information for the planning of nursing interventions involving adolescent alcohol and tobacco users and for the development of prevention and health promotion actions.
